# Comprehensive retail network dataset for bangladesh: Shop-level sales and location data

**DOI:** 10.1016/j.dib.2025.112326

**Published:** 2025-12-04

**Authors:** Md. Mahmudul Hasan Shahin, Jannatul Ferdous Swarna, Dewan Md. Farid

**Affiliations:** a Department of Computer Science and Engineering, United International University United City, Madani Avenue, Badda, Dhaka 1212, Bangladesh; b Department of Computer Science and Engineering, Southeast University, 252, Tejgaon Industrial Area, Tejgaon, Dhaka 1208, Bangladesh

**Keywords:** Geographic Information System (GIS), Route Planning, Map data, Resource planning

## Abstract

This dataset presents comprehensive retail network information for Bangladesh, collected between Feb-2022 to Oct-2024. The dataset contains detailed information for **719,817** retail outlets across all 8 administrative divisions, 64 districts, and 515 sub-districts of Bangladesh. Each outlet is identified by a unique shop code and precisely geolocated using GPS coordinates (latitude/longitude). The dataset comprises three interconnected files: (1) shop information including location, type, importance, and operational details; (2) product information covering 42 SKU variants across 5 brands and 9 product types with pricing structures; and (3) sales targets for 17 products across 613,656 shops. This multi-dimensional dataset enables spatial analysis, market segmentation, supply chain optimization, and retail performance studies across Bangladesh's retail landscape.

Specifications Table

**Table:** 

Subject	Computer Sciences
Specific subject area	Data Mining, Data Science, and Machine Learning
Type of data	CSV file
Data collection	The data collection process combined field surveys, existing company data, and expert input to create a comprehensive and reliable dataset representing Bangladesh’s retail landscape. All 8 administrative divisions of Bangladesh then district (zila), sub-district (upazila). In total coverage 64 districts and 515 sub-districts. Experts designed survey and mobile app; local surveyors trained for data collection. Nationwide in-person retail surveys conducted, collecting shop data with GPS coordinates and product details, uploaded in real-time. Sales targets set using historical data, local market analysis, and algorithm-driven forecasts, with regional manager oversight.
Data source location	United International University, Dhaka, Bangladesh
Data accessibility	Data name: Comprehensive Retail Network Dataset of Bangladesh Repository name: Mendeley Data Data identification number: 10.17632/mgzvngzng2.1 Direct URL to data: https://data.mendeley.com/datasets/mgzvngzng2/1
Related research article	

## 1 Value of the Data

The potential value of this dataset to the scientific community includes:

• **Comprehensive Retail Landscape Mapping:** This dataset provides a unique, large-scale view of Bangladesh’s retail sector, offering researchers an unprecedented opportunity to study retail distribution patterns, market penetration, and regional economic variations across an entire nation [1,2].
• **Geospatial Analysis Potential:** With precise location data for over 719,817 retail outlets, researchers can conduct advanced spatial analyses, including studies on urban development, rural-urban economic disparities, and the impact of geographical factors on retail performance [8].
• **Multi-Dimensional Performance Metrics:** The inclusion of detailed sales data across multiple product lines enables in-depth studies on consumer behaviour, product popularity variations, and pricing strategies across different regions and shop types [7].
• **Supply Chain and Distribution Network Optimisation:** The hierarchical information on distribution channels provides a rich foundation for research in supply chain management, logistics optimisation, and the study of informal economic networks in developing economies [3,4,5,6].
• **Benchmark for Retail Analytics:** This extensive dataset can serve as a valuable benchmark for developing and testing new algorithms in areas such as sales forecasting, market segmentation, and retail network optimisation, potentially leading to innovative approaches in retail analytics and business intelligence.

## 2 Background

The dataset was compiled as part of a broader initiative to gain a detailed understanding of the retail ecosystem across Bangladesh. The primary motivation stemmed from the need to systematically map and analyze the performance of retail outlets at a national scale. By capturing the exact geographic coordinates of each retail location, the data enables precise spatial referencing and supports a wide range of geospatial analyses. The inclusion of sales volumes and product-specific revenue figures was driven by the objective to monitor market activity and retail dynamics across diverse regions and product categories. Additionally, the hierarchical structure of the distribution network—encompassing contractors, collectors, and delivery routes—was mapped to understand the flow of goods from production to point-of-sale. This dataset was originally collected to support internal operational planning and optimization but also serves as a foundational resource for empirical research in retail analytics, supply chain studies, and regional economic assessment. If linked to a related research article, this dataset provides the granular, multidimensional data necessary to validate models and theories, and to extend findings through further spatial, economic, or network-based analysis (Table 1, Table 2, Table 3, Table 4, Table 5, Table 6).

**Table 1:** Demographic dimension table (shop info.csv).

Column Name	Description
**shop code**	Unique identifier for each shop
**organizer**	Source of product collection for the shop
**place nature**	Type of shop location based on geographical activity (10 types)
**place name**	Popular name of the place where the shop is located
**freq type**	Frequency rate for visiting the shop on the route
**shop type**	Type of shop based on activity (12 types), Categorical feature.
**shop style**	Type of shop based on appearance, Categorical feature.
**shop importance**	Importance of the shop to the company (3 levels), Quantitative feature.
**ss importance**	More detailed importance of the shop to the company (7 levels), Quantitative feature.
**visit target**	Target number of visits for the shop
**latitude**	Geographical latitude of the shop
**longitude**	Geographical longitude of the shop
**geometry**	Geographical point using latitude and longitude
**zila**	District where the shop is located (64 total in Bangladesh)
**upazila**	Sub-district where the shop is located (515 total in Bangladesh)
**division**	Administrative division where the shop is located (8 total in Bangladesh)

**Table 2:** Transactional fact table (product target for shop.csv).

Column Name	Description
**shop code**	Unique identifier for each shop
**prod 1 to prod 17**	Sales target for each specific product (17 different products)
**Total**	Monthly total sales target for the shop across all products

**Table 3:** Demographic dimension table on product information (product info.csv).

Column Name	Description
**sku id**	Unique identifier for each specific product variant
**sku name**	Name of the specific product variant
**product id**	Identifier for the product (can be same for multiple SKUs)
**product name**	Name of the product
**brand**	Brand of the product (5 different brands)
**type**	Type or flavor of the product (9 different types)
**packet size**	Size of the product packet (2 types)
**price a, price b, price c**	Different prices based on shop style, Retail Price.
**price 1, price 2, price 3**	Different prices based on shop importance, Retail Price.
**ingredient**	Materials and ingredients used in the product
**freq**	How frequently the product needs to be sold in a month
**in packet**	Number of items in a packet (3 types)
**total volume**	Total volume of the product
**total price**	Total price of the product, Sell Income.

**Table 4:** Product info, features statistics and types.

column	columns - type	tot value	null count	null percent	unique - values
**sku id**	int64	42	0	0	42
**sku name**	object	42	0	0	42
**product id**	int64	42	0	0	24
**product name**	object	42	0	0	24
**brand**	object	42	0	0	5
**type**	object	42	0	0	9
**packet size**	int64	42	0	0	2
**price a**	float64	42	0	0	8
**price b**	int64	42	0	0	8
**price c**	float64	42	0	0	9
**price 1**	float64	37	5	11.9	4
**price 2**	float64	24	18	42.86	4
**price 3**	float64	24	18	42.86	4
**ingredient**	float64	30	12	28.57	28
**freq**	int64	42	0	0	1
**in packet**	int64	42	0	0	3
**total volume**	float64	42	0	0	18
**total price**	float64	42	0	0	21

**Table 5:** Shop information, features statistics and types.

column	columns - type	tot value	null count	null percent	unique -values
**shop code**	object	832,881	0	0	832,881
**organizer**	object	747,714	85,167	10.23	62
**place nature**	object	721,322	111,559	13.39	10
**place name**	object	747,714	85,167	10.23	86,217
**freq type**	object	747,714	85,167	10.23	1277
**shop type**	object	747,714	85,167	10.23	12
**shop style**	object	747,714	85,167	10.23	3
**shop importance**	object	747,714	85,167	10.23	3
**ss importance**	object	747,714	85,167	10.23	7
**visit target**	float64	2585	830,296	99.69	27
**latitude**	float64	719,817	113,064	13.58	538,651
**longitude**	float64	719,817	113,064	13.58	312,967
**geometry**	object	832,881	0	0	703,324
**zila**	object	719,679	113,202	13.59	64
**upazila**	object	719,679	113,202	13.59	515
**division**	object	719,679	113,202	13.59	8

**Table 6:** Shop target, features statistics and types.

column	columns - type	tot value	null count	null percent	unique val- ues
**shop code**	object	832,881	20,850	2.44	832,881
**prod 10**	float64	735,075	118,656	13.9	5092
**prod 1, prod 2, prod 3, prod 4, prod 5, prod 6, prod 7, prod 8, prod -** **9, prod 9, prod 11, prod 12, prod 13, prod 14, prod 15, prod 16, prod - 17 columns type, tot value, null count, null percent** are same as **prod 10** & **unique values** are 885, 2341, 4615, 882, 4037, 25, 5975, 10,969, 5279, 771, 2658, 5304, 440, 4465, 3137, 789					

## 3 Data Description

This dataset offers a comprehensive view of a retail distribution network in Bangladesh, collected between Feb-2022 to Oct-2024, encompassing three interconnected aspects: product sales targets, product information, and shop demographics. The data is spread across three CSV files, each serving a distinct purpose:

1. product target for shop.csv Contains sales targets for 18 different products across 613,656 unique shops, identified by a unique shop code. This file allows for analysis of product-specific sales goals at the individual shop level.
2. product info.csv Provides detailed information about the products, including their SKU IDs, names, brands, types, pricing structures, and physical characteristics. This file offers insights into the product portfolio, including 5 brands, 9 product types, and various packaging options.
3. shop info.csv Presents demographic and geographic information about each shop in the network. It includes data on shop location (down to latitude and longitude), administrative divisions (division, zila, upazila), shop categorisation (type, style, importance), and operational details (visit frequency, organiser)

**Fig. 1:**
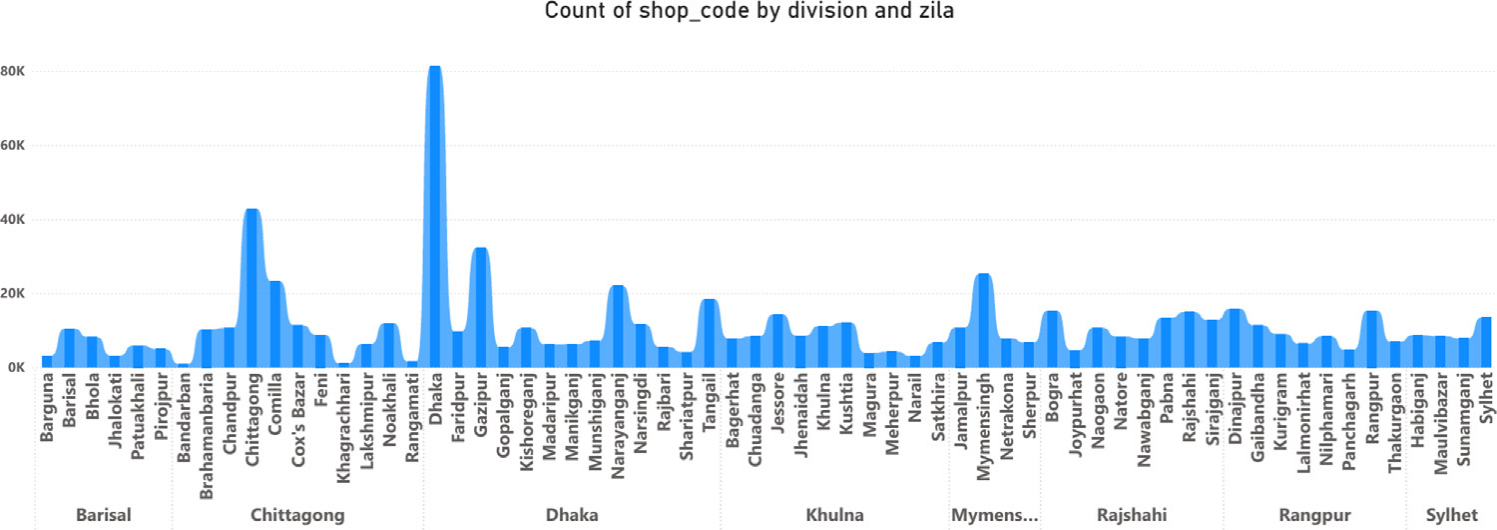
Shop count by **division** and **zila.**

In Fig. 1 One district dominates because it is the Capital of the Country, so the number of samples is higher.

**Fig. 2:**
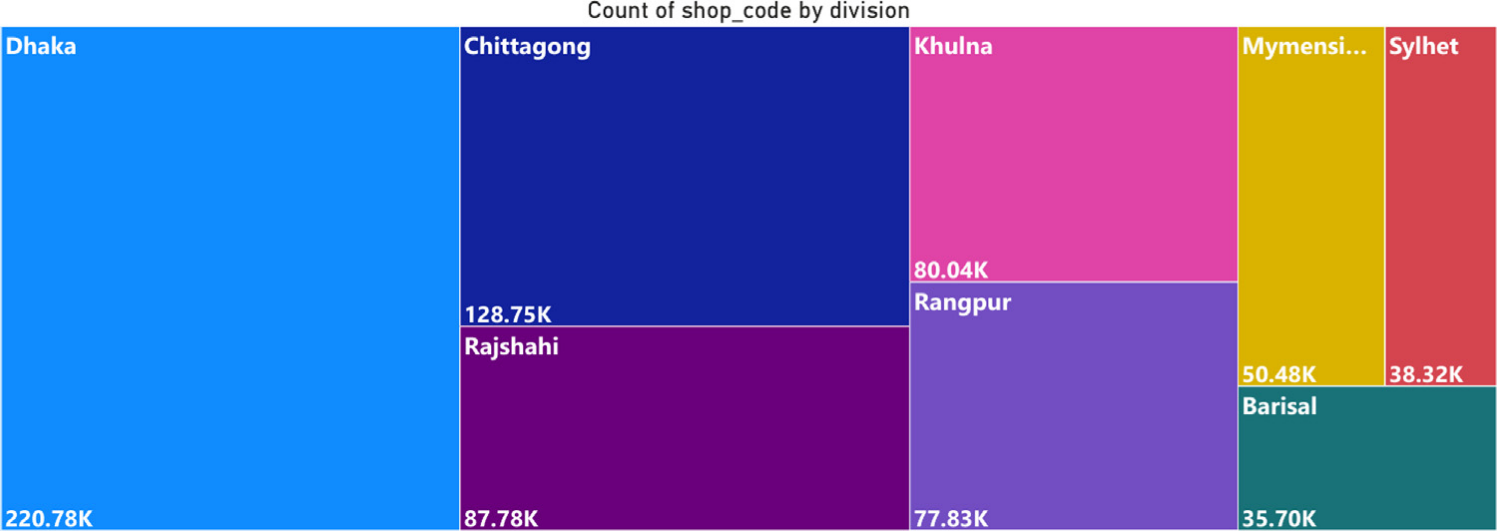
Tree map for the **division.**

**Fig. 3:**
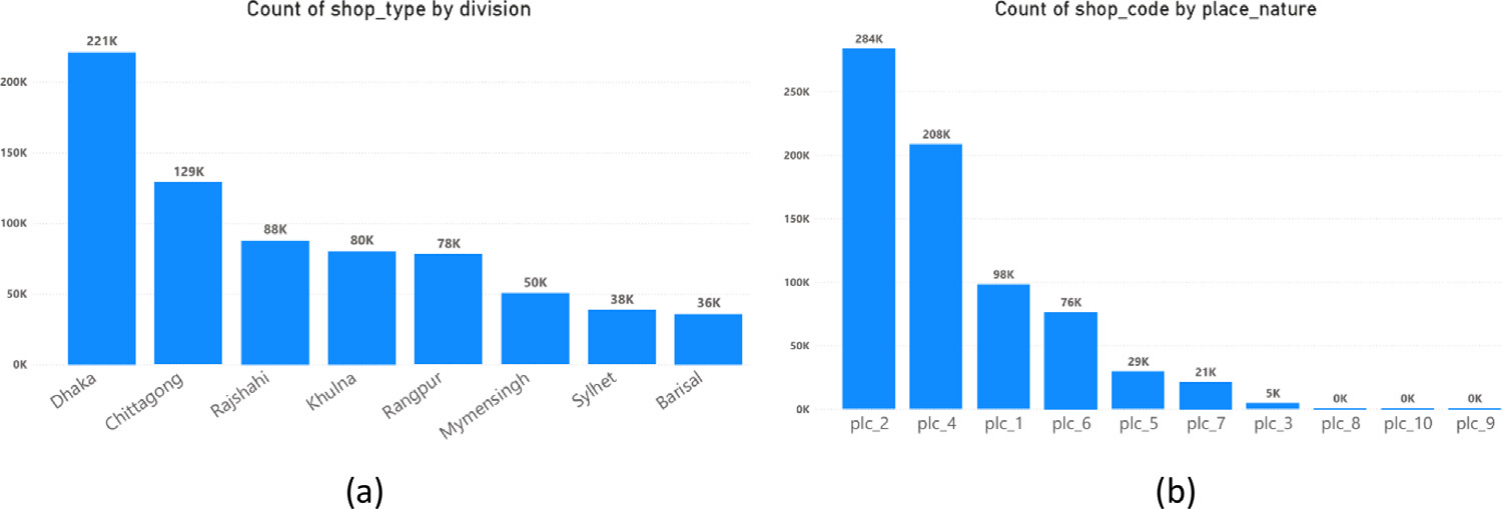
(a) Shop types on division. (b) place nature.

**Fig. 4:**
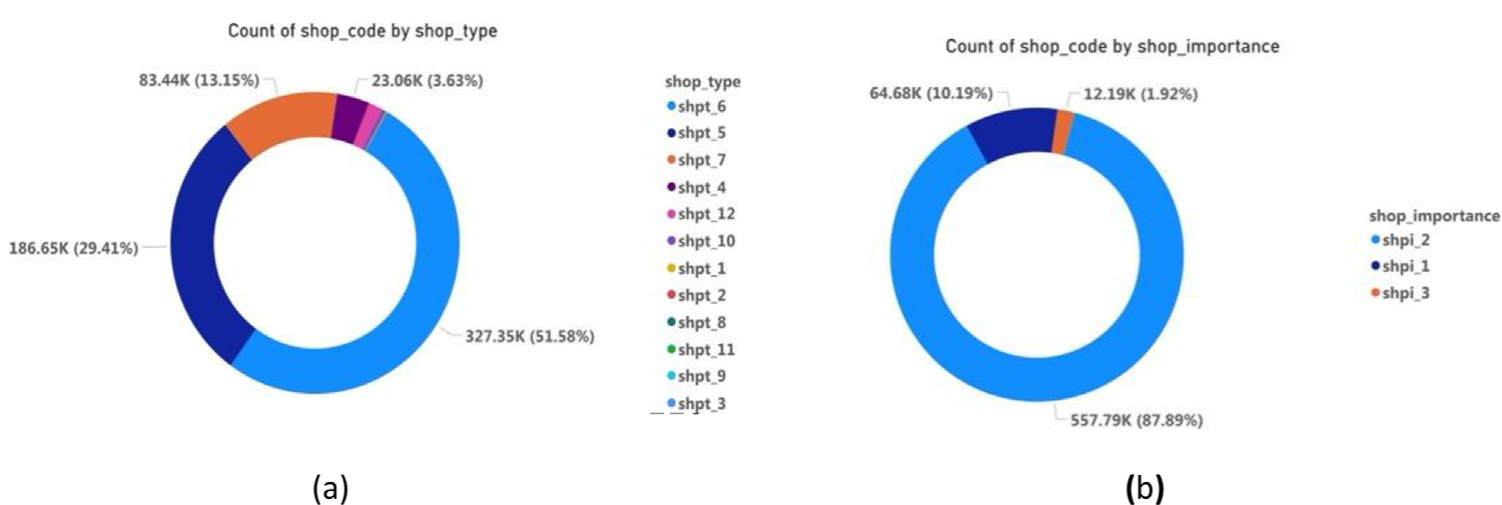
(a) Count and Share % of **Shop Type. (**b**)** Count and Share % of **Shop Importance.**

**Fig. 5:**
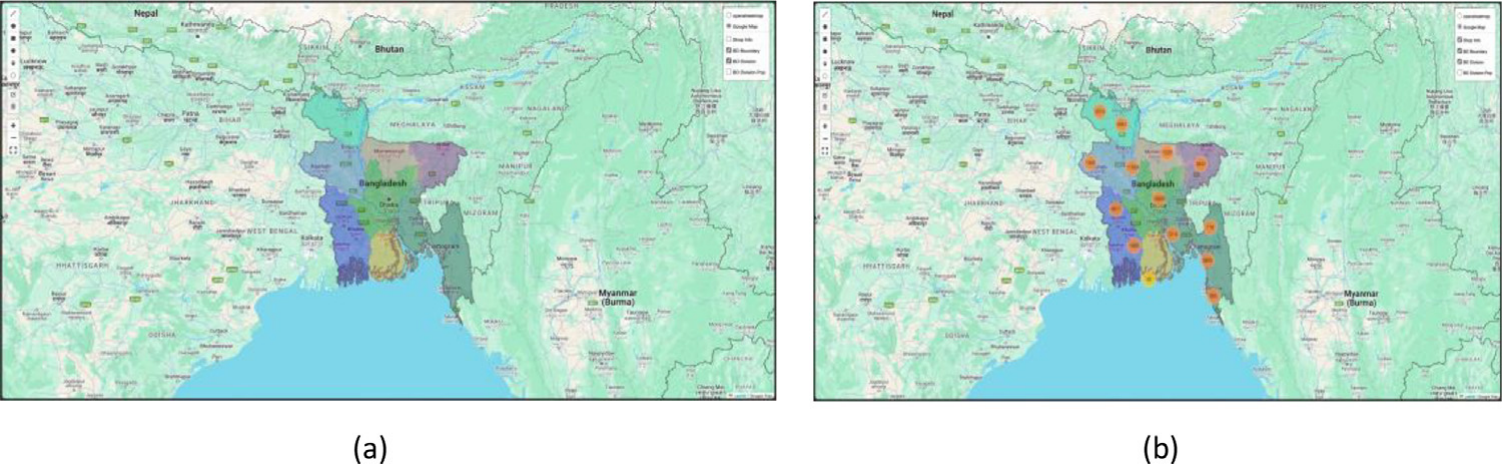
(a) Division Map. (b) Shop Marker on Map.

**Fig. 6:**
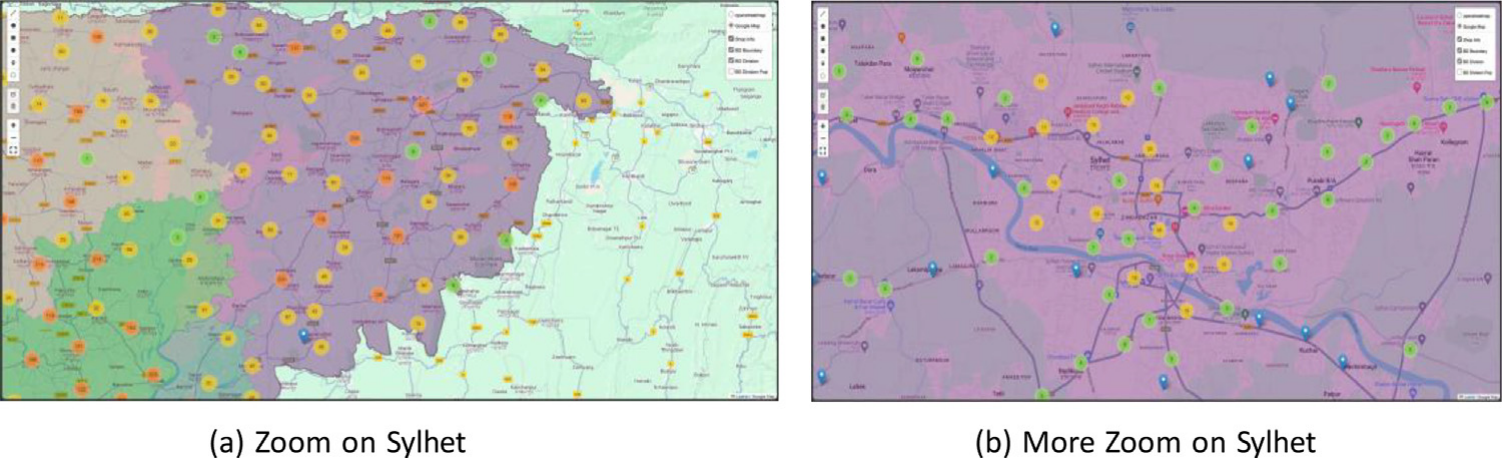
Sylhet Division Outlet.

Together, these files create a multi-dimensional picture of the retail landscape (Fig. 2,3& 4), enabling researchers to explore relationships between product characteristics, shop attributes, geographic location (Fig. 5& 6), and sales targets. This rich dataset provides a foundation for analyses ranging from market segmentation and product placement strategies to geospatial distribution patterns and supply chain optimization.

## 4 Experimental Design, Materials and Methods

**Data Collection:**

i. **Shop Information:** A nationwide retail survey was conducted in Bangladesh, encompassing all ad- ministrative levels: 8 divisions, 64 districts (Fig. 1), and 515 sub-districts. Each retail outlet was assigned a unique shop code and precisely geolocated using GPS. Surveyors classified shops according to predefined categories, including place nature (10 types based on geographical activity), shop type (12 types based on business activity), shop style (appearance-based), and importance (using 3-level and 7-level scales). The survey also captured additional metadata such as the shop’s supply source (organizer) and required visit frequency.
ii. **Product Information**: A comprehensive product catalogue was developed, assigning each variant a unique sku id. Products were categorised by brand (5 types), flavour (9 categories), packet size (2 types), and items per packet (3 types). Pricing data was collected with six price points: three based on shop style (a, b, c) and three on shop importance (1, 2, 3). The catalogue also included product composition, volume information, and established sales frequency targets for each item.
iii. **Sales Targets**: An Ensemble-Based Algorithm was developed to set sales targets, integrating multiple factors such as historical sales data, shop characteristics (e.g., type, importance, location), product attributes (e.g., brand, type, pricing), and local market potential. The algorithm combines the strengths of several individual models to enhance accuracy and handle complex, non-linear relationships within the data.
iv. **Data Processing and Validation**: Raw survey and sales data were compiled into CSV format and subjected to a rigorous cleaning process. This included removing duplicates, standardising formats, and correcting logical inconsistencies. Geospatial validation involved plotting coordinates and mapping shops to administrative divisions to ensure accuracy. Statistical analysis identified outliers in sales targets and pricing. For quality assurance, 5 % of the data was randomly selected for manual verification against original survey forms.
v. **Operational Details**: Operational details capture shop-level logistics and route management information. Key variables include visit frequency (freq type), indicating how often each shop is visited (e.g., daily, weekly); organizer, representing the source of product collection (contractor, depot, or distributor); and visit target, the planned number of visits per shop for route optimization. These variables support analyses of field operations, distribution efficiency, and network performance, as summarized in Table 2 and detailed in the “Data Collection” section.

Data Anonymisation:

All personally identifiable information related to shop owners or employees was removed from the dataset. Product names and brands were replaced with generic identifiers (e.g., prod 1, brand A) to maintain commercial confidentiality.

Quality Assurance:

Final dataset underwent logical checks, statistical analysis, and expert review to ensure completeness, accuracy, and real-world consistency.

Tools and Software:

1. **Data collection:** Custom mobile application developed for Android devices
2. **Geospatial data:** GPS-enabled devices and Google Maps API
3. **Data processing and analysis:** Python (pandas, numpy, scipy, geopandas)
4. **Geospatial analysis and visualisation:** Plotly, Dash, Matplotlib
5. **Statistical analysis:** Python
6. **Database management:** PostgreSQL with PostGIS extension for spatial data.

A systematic area-based sampling approach was employed, targeting all retail outlets within the company's distribution network across Bangladesh's 8 divisions, 64 districts, and 515 sub-districts to ensure comprehensive geographic coverage. Survey teams encountered minimal refusal rates (<2 %) as data collection was conducted through established business relationships with retailers who were part of the company's existing network. Follow-up procedures included re-visiting outlets with incomplete initial data within 48 h and cross-verification of collected information through regional supervisors to maintain data quality. Missing data was handled through multiple strategies: GPS coordinates were obtained from alternative mapping sources when unavailable, shop classification data was imputed using similar outlets in the same geographic cluster, and incomplete sales records were excluded from target calculations rather than estimated. Quality assurance involved random verification of 5 % of collected data against original survey forms and validation of geographic coordinates through mapping software to ensure spatial accuracy.

To understand and use of these data code are attached in the Github repository: github.com/shahinvx/ gis_data

This methodology ensures a comprehensive, accurate, and valuable dataset that captures the complexity of the retail network across Bangladesh, providing a solid foundation for various analytical and research purposes.

## Limitations

This dataset has several limitations that users should consider. The data represents a snapshot from a specific time period and may not reflect current market conditions or seasonal variations in retail performance. Geographic coverage, while extensive, may have gaps in remote or inaccessible areas where survey teams could not reach all retail outlets. The sales target data reflects algorithmic projections rather than actual sales figures, which may not accurately represent real market performance. Product information is anonymized, limiting brand-specific or category-specific analyses that require actual product names. Missing data issues affect approximately 10–13 % of records in key variables such as shop location coordinates and organizational details. The dataset focuses on a single company's retail network, which may not be representative of the entire Bangladesh retail landscape. Data quality may vary across different geographic regions due to varying survey conditions and local expertise of data collectors. The hierarchical categorization of shops (importance, style, type) reflects internal company classifications that may not align with standard retail industry categories. Finally, the dataset lacks temporal depth, preventing longitudinal analysis of retail trends and seasonal patterns.

## Ethics Statement

The authors have follow the ethical requirements for publication in Data in Brief and confirming that this work does not involve human subjects, animal experiments, or any data collected from social media platforms.

## CRediT Author Statement

**Md. Mahmudul Hasan Shahin:** Data collection, Data Annotation and Processing; Model validation, Writing – Original draft. **Jannatul Ferdous Swarna:** Data collection, Data Annotation and Processing; Model validation, Writing – Original draft. **Dewan Md. Farid:** Conceptualisation, Analytical reviewing, Supervision.

## Data Availability

Mendeley DataComprehensive Retail Network Dataset of Bangladesh (Original data).
